# Drifts in N-Linked Glycosylation Result in ADCC Potency Variation of Perjeta® from August 2020 to October 2021 in China

**DOI:** 10.1155/2022/7868391

**Published:** 2022-04-30

**Authors:** Bing Yao, Di Zhang, Weirong Cao, Gaoqiang Yang, Wenbin Li, Xiwu Hui, Pingkai Ouyang, Guoguang Chen, Boning Liu

**Affiliations:** ^1^School of Biotechnology and Pharmaceutical Engineering, Nanjing Tech University, Nanjing, China; ^2^CSPC Zhongqi Pharmaceutical Technology (SJZ) CO., LTD., Shijiazhuang, China; ^3^Bioworkshops (Suzhou) Limited, Suzhou, China

## Abstract

The proposed biosimilar candidate needs to demonstrate biosimilarity with reference products, and the quality target product profile and biosimilarity assessment criteria are prerequisite, which should be based on extensive characterization of the reference products. In this study, 13 lots of China-sourced pertuzumab (trademark: Perjeta®), with an expiration date from 2020 to 2021, were comprehensively characterized. Despite the consistency of purity, drifts in N-glycan profile were observed, which resulted in the variation of antibody-dependent cellular cytotoxicity (ADCC) activity. In detail, four parametric curves of ADCC activity of the reference product were unparalleled, and the maximum response value was highly related to the content of %afucose than half-maximal effective concentration (EC_50_). As ADCC is a potential critical quality attribute of Perjeta®, the glycosylation of Perjeta® and its biosimilars should be tightly monitored and controlled.

## 1. Introduction

Biosimilars are biological products that are highly similar to the previously authorized reference products, and they have no clinically meaningful differences. The US Food and Drug Administration (FDA), the European Medicines Agency (EMA), the World Health Organization (WHO), and other local drug regulatory authorities, including China National Medical Products Administration (NMPA), have published a number of guidelines for the development of biosimilars, which could be used to further clarify clinical and nonclinical evidences [1–3]. Minor changes in the manufacturing process may cause drifts in the quality of biological products. Therefore, to ensure the consistency between processes and products, a robust quality management system is urgently required [4, 5]. Evaluating the similarity of structural and functional properties is crucial to indicate whether the existing differences can influence clinical outcomes. In order to ensure that the full spectrum of product variability could be accurately captured, manufacturers should gather multiple batches of reference products throughout the biosimilar development period [6]. Considering the inherent heterogeneity among protein products and the expected lot-to-lot differences derived from manufacturing processes, the FDA guideline has recommended that manufacturers should collect at least 10 lots of reference products^[1]^.

Pertuzumab, a full-length humanized recombinant IgG1(*κ*) monoclonal antibody, targets human epidermal growth factor receptor 2 (HER2) by binding to a different epitope (domain II) with trastuzumab (domain IV), preventing the dimerization of HER2 with other members of the HER family (HER1, HER3, or HER4). Pertuzumab results in a more complete inhibition of the HER2 axis when it is combined with trastuzumab. In addition, pertuzumab activates antibody-dependent cellular cytotoxicity (ADCC), which is related to Fc*γ* receptor IIIa binding and glycosylation [7]. Perjeta® was approved by the United States FDA (2012), EMA (2013), and NMPA (2018) in combination with trastuzumab and chemotherapy for the treatment of patients with HER2-positive metastatic breast cancer.

For the development of biosimilars of pertuzumab, 13 lots of China-resourced Perjeta® with an expiration date from August 2020 to October 2021 were collected intensively for the characterization of physicochemical and functional attributes. The state-of-the-art and orthogonal methods were chosen to analyze the size, charge, and unpaired cysteine variants of pertuzumab. As reported previously, an unpaired cysteine variant of Cys23/Cys88 in one or both light chains of pertuzumab could be identified, which was proved to be related to the antiproliferation effect, and it was estimated to have a reduced potency of ~50% compared with the native Fab [8]. N-linked glycosylation of the crystallizable fragment (Fc) of the immunoglobulin class G (IgG) is a well-known attribute that is significantly associated with Fc-mediated antibody effector functions, such as ADCC and complement-dependent cytotoxicity (CDC). Afucosylation content dramatically enhances the ADCC activity by increasing the binding affinity between the antibody and Fc*γ* receptor IIIa [7, 9, 10]. The drifts in N-glycan-related quality attributes have been extensively discussed in previous studies (e.g., trastuzumab) [4, 11, 12]. However, no previous research has demonstrated that the quality attributes of pertuzumab have drifted.

In the present study, we found that the size, charge, unpaired cysteine variants, and bioactivity were highly consistent among all batches of pertuzumab, while two sequential drifts of glycosylation were observed, and the same drifts were also detected in Fc*γ*-IIIa binding affinity and ADCC activity assay. Pertuzumab could be divided into three groups according to the drift. Importantly, we found that the drifts in ADCC-related quality attributes of pertuzumab were related not only to EC_50_ but also to maximum response. Here, we provided the characterization data and representative profiles for Perjeta® and discussed the potential consequences of the drifts.

## 2. Materials and Methods

### 2.1. Materials

A total of 13 batches of Perjeta® (420 mg/vial) were purchased from China, and the corresponding batch numbers and expiration date are shown in Table 1.

The CellTiter-Glo Luminescent and Bio-Glo Luciferase Assay Reagent was obtained from Promega (Madison, WI, USA). Goat anti-human IgG- (Fc specific) peroxidase antibody, sodium dodecyl sulfate (SDS), n-ethyl maleimide (NEM), sodium cyanoborohydride, 2-aminobenzamide (2-AB), and 3,3′,5,5′-tetramethylbenzidine (TMB) were purchased from Sigma-Aldrich (St. Louis, MO, USA). HER2 antigen was purchased from Sino Biological Co., Ltd. (Beijing, China). The PNGase F kit was obtained from New England Biolabs (Ipswich, MA, USA). The Measure-iT™ Thiol Assay kit was purchased from Thermo Fisher Scientific (Waltham, MA, USA). FabALACTICA (IgdE) was obtained from Genovis (Lund, Sweden). TSKgel FcR-IIIA-NPR column was obtained from Tosoh Bioscience (Tokyo, Japan).

### 2.2. Cell Lines and Cell Culture

MDA-MB-175VII cells were obtained from the American Type Culture Collection (ATCC; Manassas, VA, USA) and were incubated at 37°C in a L-15 medium (Hyclone, Logan, UT, USA), containing 10% fetal bovine serum (FBS) (Gibco, New York, NY, USA). SK-BR-3 cells were obtained from the ATCC and incubated at 37°C, with 5% CO_2_ in a Roswell Park Memorial Institute- (RPMI-) 1640 medium (Sigma-Aldrich) containing 10% FBS (Gibco). Jurkat-CD16a-Fc*ε*RI*γ*-NFAT-luc reporter cell line was purchased from the National Institutes for Food and Drug Control (NIFDC) and incubated at 37°C, 5% CO_2_ in a RPMI-1640 medium (Sigma-Aldrich) with 10% FBS (Gibco), 500 *μ*g/mL G418(Gibco), 1 mM sodium pyruvate (Gibco), and 1 *μ*g/mL puromycin (Gibco).

### 2.3. Purity Assay: Size and Charge Variants

Size variants of pertuzumab reference products were detected by size-exclusion chromatography (SEC) and capillary electrophoresis-sodium dodecyl sulfate (CE-SDS). 100 *μ*g samples were directly injected into a TSKgel G3000 SW_XL_ analytical column (Tosoh Bioscience; 0008541, 5 *μ*m/7.8 mm × 300 mm) at 25°C, which was connected to a Waters high-performance liquid chromatography (HPLC) system (Waters Corporation, Milford, MA, USA). Mobile phase consisted of 200 mM potassium dihydrogen phosphate and 150 mM potassium chloride (pH 6.8). The flow rate was 0.5 mL/min, which was run for 30 min. The elution profile was monitored at 280 nm. CE-SDS was used to separate denatured protein size variants under reducing or nonreducing conditions. For reducing CE-SDS (rCE-SDS), *β*-mercaptoethanol was added to the protein denaturation solution to reduce the disulfide bonds. For nonreducing CE-SDS (nrCE-SDS), NEM was added to the denaturation solution to prevent protein degradation. After denaturation, rCE-SDS samples were injected into PA800 Plus, and nrCE-SDS samples were injected into Maurice.

Charge variants of pertuzumab reference products were separated by ion exchange chromatography (IEX) and imaged capillary isoelectric focusing (icIEF). Samples were separated on a ProPac WCX-10 analytical column (Thermo Fisher Scientific; 054993, 10 *μ*m/4.0 × 250 mm), the column temperature was controlled at 35°C, and the salt gradient elution was performed. Mobile phase A consisted of 10 mM phosphate buffer (pH 6.8), and mobile phase B consisted of mobile phase A and 400 mM sodium chloride. The flow rate was 1.0 mL/min and run for 40 min, and the gradient elution from 0 to 30 min with mobile phase B changed from 5% to 25%. The fractions of the charge variants were collected and injected into the mass spectrometry for further characterization. The charge heterogeneity was also analyzed by icIEF. Then, 20 *μ*g of sample was taken and added into 90 *μ*L icIEF mixture. The mixture contained 35 *μ*L 1% methyl cellulose, 4 *μ*L Pharmalyte 3–10, 40 *μ*L 10 M urea, 2 *μ*L 500 mM arginine, pI Marker 0.5 *μ*L each, and 8 *μ*L ultrapure water. Samples were focused at 1500 V for 1 min and 3000 V for 8 min.

### 2.4. Analysis of Unpaired Cysteines

Three methods were used for the analysis of unpaired cysteine: hydrophobic interaction chromatography (HIC), reversed-phase high-performance liquid chromatography (RP-HPLC), and Measure-iT™ Thiol assay. The samples were digested with IgdE after removing the C-terminal lysine with carboxypeptidase B (CpB). The Fab and Fc domains were separated by HIC using a Polypropyl Aspartamide column (PolyLC, 104PR0315, 3 *μ*m/4.6 × 100 mm). Solvent A consisted of 1.6 M ammonium sulfate and 20 mM potassium phosphate (pH 6.05), and solvent B consisted of 20 mM potassium phosphate (pH 6.05). The analytes were separated with a gradient from 0 to 100% solvent B for 0-35 min. The column temperature was maintained at 25°C with a flow rate of 0.8 mL/min. The elution profile was monitored at 280 nm [8].

The samples were reduced with DTT in the absence of denaturants, and then, the light chain and heavy chain were separated by RP-HPLC using a BioResolve RP mAb Polyphenyl column (Waters Corporation; 186008945, 2.7 *μ*m/2.1 × 100 mm). Solvent A consisted of 0.12% formic acid in water, and solvent B consisted of 0.10% formic acid in acetonitrile. The analytes were separated by 20-40% solvent B during 3-18 min. The column was maintained at 60°C with a flow rate of 0.2 mL/min. The elution profile was monitored at 214 nm.

The free thiol groups in the samples were also determined by a Measure-iT thiol assay kit according to the manufacturer's instructions.

### 2.5. Fab-Effector Functional Bioassays

The HER2 binding activity was analyzed by using an enzyme-linked immunosorbent assay (ELISA). First, a 96-well plate was coated with 125 ng/well HER2 antigen at 2-8°C for 18 h, the plate was blocked with blocking buffer for 2 hat room temperature, and then, 100 *μ*L/well of diluted reference standard or samples (from 0.17 to 10,000 ng/mL) was transferred into separate wells. After sample loading, the plate was incubated at 25°C for 2 h. Anti-human IgG-peroxidase was prepared at a dilution of 1 : 300,000, and 100 *μ*L/well diluted secondary antibody was added. After the addition of TMB substrate, optical density (OD) was measured at a wavelength of 450/650 nm on a plate reader. The relative HER2 binding activity was equal to the percentage of EC_50_ of the reference standard to the EC50 of the tested sample.

Inhibition of cell proliferation was determined by CellTiter-Glo luminescent cell viability assay. First, MDA-MB-175VII cells at a density of 20,000 cells/well were incubated overnight, and then, different concentrations (0.0298–500,000 ng/mL) of pertuzumab were incubated for 4 days at 37°C in a L-15 medium containing 1% FBS. The relative number of viable cells was quantified by measuring the luminescence using a SoftMax GXP software to calculate the relative antiproliferation potency.

### 2.6. Glycosylation Profiling Assay

N-glycan profile was analyzed using hydrophilic interaction liquid chromatography (HILIC). Denaturing and reducing reagents were added to 500 *μ*g samples, incubated at 100°C for 10 min, and treated with 2*μ*L PNGase F to release N-linked oligosaccharides at 37°C for 1 h. After the released oligosaccharides were precipitated with precooled ethanol, the supernatant was dried and labelled with 2-AB for 3 h at 65°C. The reaction mixtures were injected into an ACQUITY UPLC glycan BEH amide column (Waters Corporation; 186004742, 1.7 *μ*m/2.1 mm × 150 mm) and separated at a flow rate of 0.5 mL/min with mobile phase A (50 mM ammonium acetate) and mobile phase B (100% acetonitrile). The signal was detected using a fluorescence detector at the Ex 265 nm/Em 425 nm.

### 2.7. Fc-Effector Functional Bioassays

An affinity column (Tosoh Bioscience; 0023513, 4.6 mm × 75 mm) packed with Fc*γ*RIIIa immobilized resin was used. Then, 20 *μ*g of samples was loaded onto the column and separated at a flow rate of 1.0 mL/min with mobile phase A (50 mM citric acid at pH of 6.5) and mobile phase B (50 mM citric acid at pH of 4.0) for 30 min. The elution profile was monitored at 280 nm.

The ADCC assay was performed using Jurkat-CD16a-Fc*ε*RI*γ*-NFAT-luc reporter cell lines as effector cells. SKBR3 cells were incubated at a density of 2E4 cells/well overnight at 37°C in a 96-well plate, and then, effector cells and serial dilution antibodies were added (2.3–45,000 ng/mL) to the plate. The ratio of effector cells : target cells was 7.5 : 1. After 6 h of induction at 37°C, Bio-Glo luciferase reagent was added and luminescence activity was determined using a SpectraMax L microplate reader. The dose response curves were fitted with a four-parameter model, and parallelism was calculated using the SoftMax GXP software.

### 2.8. Software and Equipment

The HPLC system, ultraperformance liquid chromatography (UPLC) system, and the Empower 3 software were obtained from Waters Corporation. The icIEF instrument (Maurice) was obtained from ProteinSimple, Inc. (San Jose, CA, USA). The microplate reader and the SoftMax Pro 7.0.3 were purchased from Molecular Devices, Inc. (San Jose, CA, USA). The CE system (PA800 Plus) was purchased from AB SCIEX (Framingham, MA, USA).

## 3. Results

### 3.1. Perjeta® Has a High Consistency in Size, Charge, Unpaired Cysteine Variants, and Biological Activity

**Figure 1:**
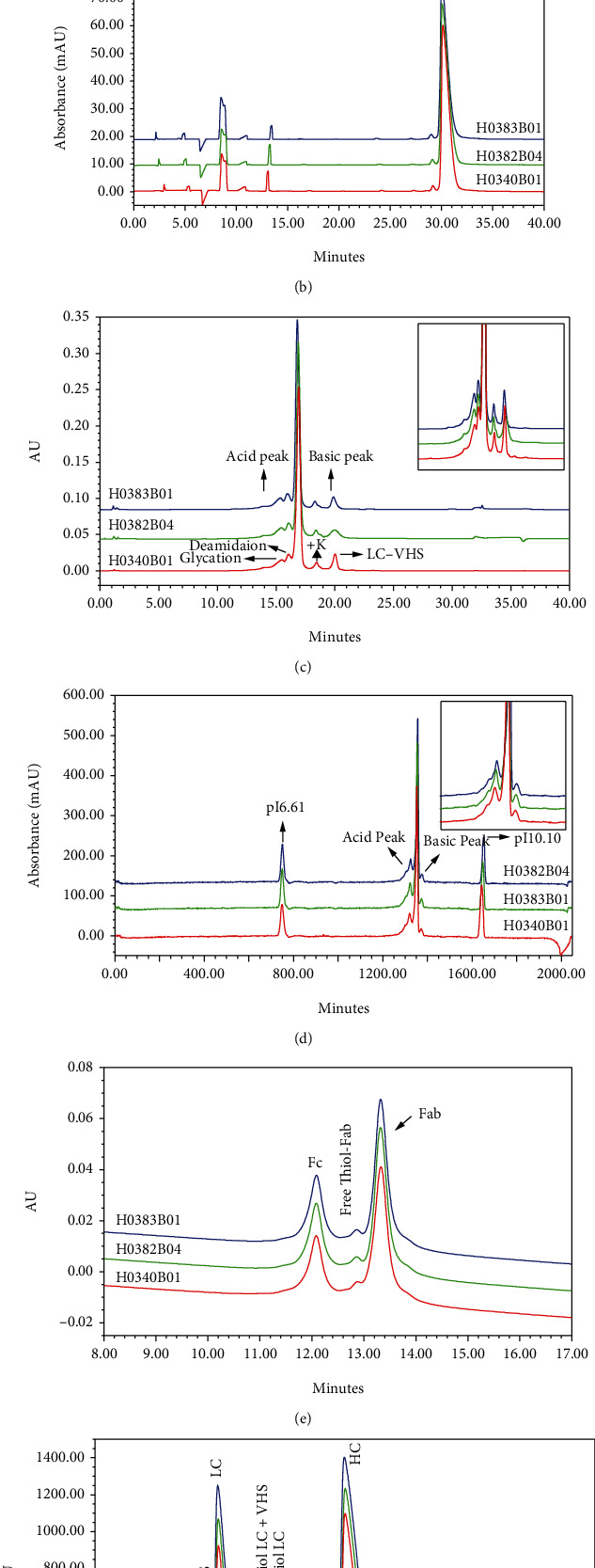
Quality attributes of Perjeta®, which are consistent among all the monitored batches. Size variant profiles assessed by (a) SEC-HPLC and (b) NR CE-SDS; charge variant profiles assessed by (c) IEX-HPLC and (d) icIEF; unpaired cysteine variant profiles assessed by (e) HIC-HPLC: the samples were digested by CpB and IgdE enzyme. (f) RP-HPLC: the samples were reduced by DTT. Three typical batches of Perjeta® are displayed, including one batch (H0382B04) from the high-afucose level group, one batch (H0340B01) from the medium-afucose level group, and one batch (H0383B01) from the low-afucose level group. HMW: high molecular weight impurities; LMW: low molecular weight impurities.

In the present study, state-of-the-art methods were applied for physicochemical characterization of Perjeta® with expiration date from August 2020 to October 2021, and the results confirmed that a high degree of consistency was observed for size, charge, and unpaired cysteine variants throughout all the lots that were monitored. The detection results of size variants, such as SEC-HPLC and nrCE-SDS were 99.72% ± 0.02% and 97.87% ± 0.19%, respectively (Figures 1(a) and 1(b) and Supplementary Table 1). The main peak percentage of charge variants, such as IEX-HPLC and icIEF, was 66.30% ± 0.55% and 69.02% ± 0.92%, and the acid peak contents of IEX-HPLC and icIEF were 20.28% ± 1.06% and 26.47% ± 0.85%, respectively (Figures 1(c) and 1(d) and Supplementary Table 1). Mass spectrometry analysis of the fractions collected by IEX-HPLC indicated that the main posttranslational modified acidic variants were glycation, carboxymethyllysine (CML), and deamidation. The basic fractions were composed of C-terminal lysine variant, glycine loss with proline amidation, and light chain (LC) with truncated signal peptide VHS variant. The binding activity and antiproliferation activity of each fraction suggested that there was no significant difference from the main peak (data were not shown).

To assess the contents of unpaired cysteine variants in pertuzumab, three methods were used. Free thiols were measured using Measure-iT™ Thiol Assay Kit and showed a reactive free sulfhydryl content of 0.24 ± 0.04 moles per mole protein of pertuzumab under denaturing conditions. Free thiols were below the quantitation limit in all test batches in the absence of denaturants, indicating that the free thiols (i.e., unpaired cysteines) were buried and inaccessible to Measure-iT™ thiol quantitation reagent in pertuzumab molecules under nondenaturing conditions (see Supplementary Table 1) [13, 14]. Analysis of pertuzumab by HIC after CpB and IgdE digestion revealed an additional peak between the Fc and Fab peaks, which was identified as a Fab variant containing unpaired cysteine residues [8]. The results of HIC showed that the contents of free sulfhydryl variants in all 6 typical batches were very close, ranging from 10.22% ± 0.17% (Figure 1(e) and Supplementary Table 1). The results of reduced RP-HPLC indicated that the percentage of unpaired cysteine on the LC was 15.82% ± 0.17% (see Supplementary Table 1), and the LC VHS with unpaired cysteine was separated in front of LC with unpaired cysteine (Figure 1(f)).

HER2 binding and antiproliferation assays were performed to evaluate the Fab-related activities of pertuzumab. The results revealed that the Fab-related bioactivities of the reference products were consistent and were all within an acceptable range. The antiproliferation activity of the reference products was between 95.69% ± 13.02%, the HER2 binding activity was 106.31% ± 8.99%, and deviations were within an acceptable range (see Supplementary Table 2).

### 3.2. Drifts in %Afucose and %Galactose Were Observed during the Monitoring Period of Perjeta®, and the Results of the Fc*γ*RIIIa Affinity Further Proved the N-Glycan Drifts

**Figure 2:**
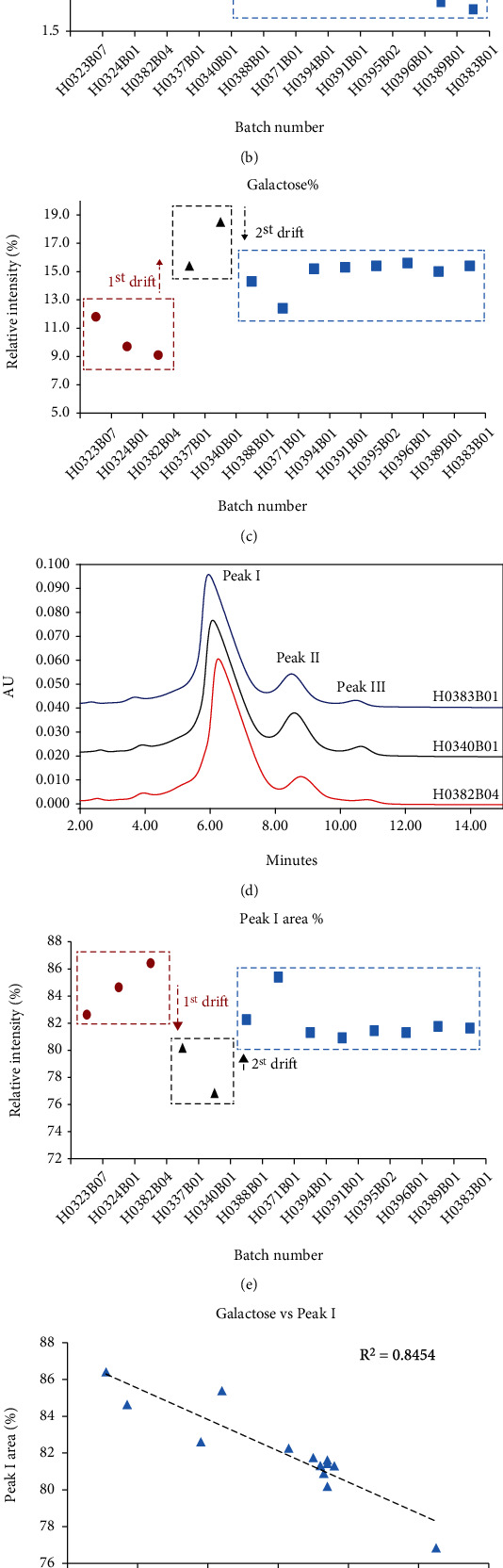
Drifts in N-linked glycosylation are illustrated, and the reference products can be divided into three groups according to the drifts in %afucose and % galactose. (a) N-glycan profile of the three typical batches of Perjeta®. (b) Scatter plot shows the drifts in %afucose and (c) %galactose. Fc*γ*RIIIa affinity analysis further proved drifts in the N-glycan. (d) Fc*γ*RIIIa affinity chromatogram of three typical batches of Perjeta®. (e) The percentage of peak I area shows an opposite trend to %galactose. The contents of %afucose and %galactose negatively correlated with the percentage of peak I area, and %galactose exhibited a stronger correlation with Fc*γ*RIIIa affinity. (f) Correlation analysis between the percentage of peak I area and the %galactose. (g) Correlation analysis between the percentage of peak I area and the N-glycan (%galactose + %afucose).

The glycoform profiles of pertuzumab reference products were detected by HILIC-UPLC, and identical glycoform species existed in all products (Figure 2(a)). Among 13 batches of reference products collected intensively in the past two years, relatively larger variations in glycoform distribution were observed. The relative abundance rates of afucosylated and galactosylated glycoform in the pertuzumab were 2.58% ± 0.84% and 14.08% ± 2.63%, respectively. The reference products could be divided into three groups according to the drift of %afucose. The batches involved in the high-level group were H0323B07/H0324B01/H0382B04 with the %afucose between 3.7% and 4.2%; the medium-level group referred to batches of H0337B01/H0340B01 with %afucose between 2.4% and 2.6%, and the low-level group referred to other 13 lots of pertuzumab, with the %afucose between 1.8% and 2.3% (Figure 2(b)). The reference products could be divided into the same three categories according to the %galactose content (Figure 2(c)). The effects of the N-glycan drifts were further investigated by Fc functional assays.

Affinity chromatography (AC) is a new method aiming to analyze the affinity of Fc domain and the receptor [15]. Further study of the relationship between the oligosaccharide content and AC-HPLC peak area may provide more direct evidence for correlation among glycoforms, Fc*γ*RIIIa binding affinity, and ADCC activity. The AC-HPLC separated pertuzumab into three major peaks (peaks I-III), the later-elution peaks represented a higher Fc*γ*RIIIa affinity, while the earlier-elution peaks represented a lower Fc*γ*RIIIa affinity (Figure 2(d)). The percentage of peak I area increased from 76.8% to 86.4%, accompanying with the reduction of affinity (see Supplementary Table 2). The relationship between the percentage of peak I area and the monosaccharide content was studied. The results indicated that the contents of %afucose and %galactose of pertuzumab were positively correlated with the affinity of Fc*γ*RIIIa, and %galactose exhibited a stronger correlation with Fc*γ*RIIIa affinity (Figures 2(f) and 2(g)). The relative percentage of peak area was plotted against its batch number, which was ranked according to the content of %afucose, and the Fc*γ*RIIIa affinity exhibited a similar trend with %galactose (opposite trend with the percentage of peak I area, Figure 2(e)). The results revealed that the reference product could be divided into the three categories according to the Fc*γ*RIIIa affinity, as we discussed earlier.

### 3.3. Difference in ADCC Activity of Pertuzumab Was Caused by the Drifts of N-Glycan, and the Maximum Response Was Further Associated with the %Afucose than the EC_50_

**Figure 3:**
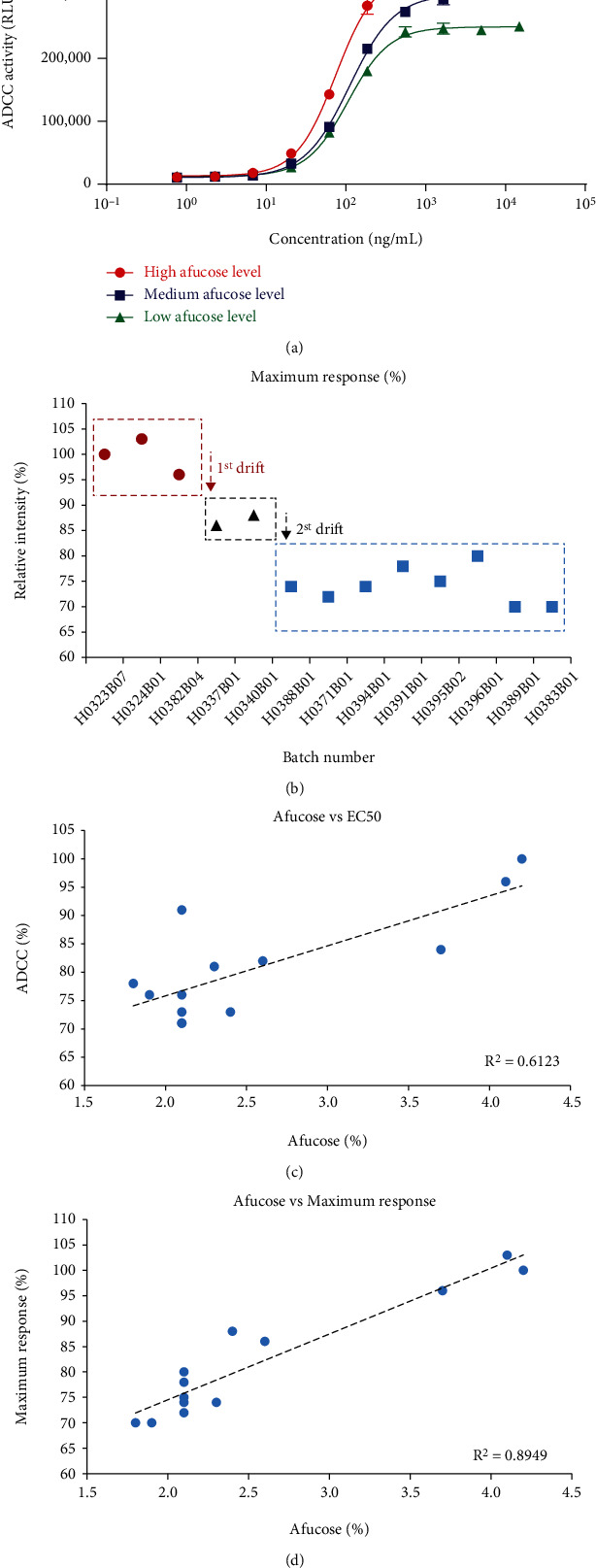
The influences of the drifts in %afucose on the ADCC activity. (a) ADCC activity of three typical batches of Perjeta® based on reporter gene assay, in which curves are unparalleled. (b) Relative maximum response shows the same drift trend as %afucose. (c) Correlation analysis between the %afucose and ADCC relative EC_50_. (d) Correlation analysis between the %afucose and the relative maximum response. The correlation between %afucose and %maximum response is higher than the correlation between %afucose and EC_50_.

As two sequential drifts of glycosylation were observed, their effects on the biological properties of Perjeta® were further assessed. Reporter-gene assay was employed to evaluate changes in core fucosylation on ADCC activity. As expected, batches of pertuzumab with a higher afucosylation showed a stronger ADCC potency. It was noteworthy that the maximum response of the dose-response curve of ADCC between batches was elevated by nearly 1.5-fold, and the relative maximum response was 70-103% (Figure 3(a) and Table 1), while the upper asymptote of the same antibody was mainly close. According to the above-mentioned results, we could conclude that the difference in ADCC activity of pertuzumab was caused by the drifts of N-glycan.

The results of the scatter plot of the maximum response of ADCC showed that the drift trend was the same as that of %afucose and divided Perjeta® into the same three groups as %afucose (Figure 3(b)). Correlation analysis showed that there was a higher correlation between %afucose and %maximum response, rather than EC_50_, which could be due to unparalleled curves (Figures 3(c) and 3(d)). We additionally calculated the parallelism among the curves using the parallel line analysis (PLA) via the SoftMax Pro 7.0.3 software. When *F*-prob was ≥0.05, the two curves were considered as parallel [16]. The parallelism of the remaining 12 batches of Perjeta® was calculated with H0323B07 (%afucose = 4.2%) as reference. The results showed that only the H0324B01 (%afucose = 4.1%) and H0382B04 (%afucose = 3.7%) batches were parallel with the reference, they all belonged to the high-level group, and the other 10 batches of Perjeta® belonged to the medium- or low-level group, in which fitting curves were judged as unparalleled.

## 4. Discussion

Due to the complexity of the structure and function of the monoclonal antibodies, even if the manufacturing process is well-controlled, hundreds of variants still exist [17]. Minor changes in the production process may lead to inconsistency and drifts in target quality attributes. It is a main challenge to ensure the consistency of the glycoform distribution lot to lot. Although manufacturers strive to avoid related changes in quality attributes, such changes are typically inevitable. Pertuzumab has a high degree of consistency in size, charge, unpaired cysteine variants, and biological activity, while drifts in the quality attributes could be related to N-glycan.

N-glycan profile is a critical quality attribute of biosimilar and is well-known to affect ADCC activity. Certain changes in monosaccharide located on the structure of oligosaccharide, especially in the core fucose or terminal residues, have been shown to affect Fc effector function, clearance, and immunogenicity, directly influencing the overall therapeutic efficacy [18, 19]. During a three-year follow-up of a phase 3 clinical study, Pivot et al. found that the event-free survival rate of Herceptin®-treated patients exposed to batches with a lower ADCC activity was significantly reduced, which supported the relationship between ADCC activity and clinical outcomes [20, 21]. The present research revealed that changes in N-glycan (%afucose and %galactose) could also cause changes in the affinity of pertuzumab to Fc*γ*RIIIa.

AC is able to separate antibodies according to their affinity to the Fc*γ*RIIIa receptor, accompanying with a higher resolution compared with surface plasmon resonance (SPR) or biolayer interferometry (BLI) method [22]. Correlation analysis showed that %galactose of pertuzumab exhibited a stronger correlation with Fc*γ*RIIIa affinity than %afucose. However, the effects of galactosylated glycoform on Fc*γ*RIIIa affinity have still remained elusive [23]. A previous study suggested that the terminal galactose could reduce the conformational entropy of CH2 domain and facilitate the binding of Fc to Fc*γ*RIIIa [15].

Our research also found that when %afucose varied from 1.8% to 4.2%, the relative maximum response changed from 70% to 103%, which was mainly close. The phenomenon was reported by other scholars using cell-based ADCC assay compared with reporter-gene assay [24, 25], indicating that it is not method-dependent. The phenomenon may be related to the nonparallelism of the dose-response curve of ADCC. The EC_50_ cannot represent the actual ADCC activity of the pertuzumab. The parallelism analysis of 4-parameter curves of ADCC showed that when the batch with the highest %afucose was taken as the reference standard, only the high-level group of the other 12 batches of pertuzumab was parallel to the reference standard. The parallelism analysis enables a user to establish parallelism if the biological response to two substances is similar or if two biological environments give similar dose-response curves to the same substances. Testing of parallelism between dose-response curves of reference standard and test sample is a prerequisite to calculate the relative potency of a compound. Only on the basis of established parallelism, calculating relative biological activity of test sample according to EC_50_ could be scientific and reasonable [26]. Therefore, when the curve is unparalleled, it is not sufficient to use EC_50_ to calculate ADCC activity.

During Perjeta's BLA application, both the FDA and EMA required ADCC activity to be included in the control strategy [27, 28]. According to the results of previous studies and our research, ADCC activity is closely correlated to the %afucose, and afucosylation could enhance ADCC activity by improving IgG binding to Fc*γ*RIIIa. Considering the nonparallelism of the dose-response curve of ADCC of pertuzumab, in the development of biosimilars of pertuzumab, our findings indicated that the level of %afucose should be involved in the release testing, rather than ADCC activity, and reasonable criteria should be provided based on the quality range of multiple batches of reference products.

## Figures and Tables

**Table 1:** Data of Perjeta® and N-glycan-related characterization results.

Group	Batch	Expiry date	N-glycan profile		ADCC (%relative activity)		
			%Afucose	%Galactose	EC_50_	Maximum response	*F*-prob
High	H0323B07	Aug-20	4.2	11.8	100	100	RS
	H0324B01	Aug-20	4.1	9.7	96	103	0.609
	H0382B04	Aug-21	3.7	9.1	84	96	0.129

Medium	H0337B01	Oct-20	2.6	15.4	82	86	4.8*E*-6
	H0340B01	Nov-20	2.4	18.5	73	88	4.0*E*-6

Low	H0371B01	May-21	2.1	12.4	91	72	1.1*E*-5
	H0388B01	Sep-21	2.3	14.3	81	74	1.3*E*-4
	H0394B01	Sep-21	2.1	15.2	73	74	1.6*E*-11
	H0391B01	Sep-21	2.1	15.3	71	78	9.4*E*-12
	H0389B01	Sep-21	1.9	15.0	76	70	3.6*E*-8
	H0383B01	Sep-21	1.8	15.4	78	70	1.7*E*-8
	H0395B02	Oct-21	2.1	15.4	76	75	2.4*E*-10
	H0396B01	Oct-21	2.1	15.6	71	80	9.1*E*-10

Mean (*n* = 13)			2.6	14.1	80.9	82.0	NA
SD (*n* = 13)			0.8	2.6	9.5	11.5	
RSD (%)			30.8	18.4	11.7	14.0	

## Data Availability

The table and the figures used to support the findings of this study are included within the article and the supplementary information file (s).

## Supplementary Materials

Supplementary Materials Supplemental Table 1: information and characterization related to purity results of Perjeta. Supplemental Table 2: information and characterization related to activity results of Perjeta.
