# Supplementary material for: Drifts in N-Linked Glycosylation Result in ADCC Potency Variation of Perjeta® from August 2020 to October 2021 in China
Source: Biomed Res Int. 2022 Apr 30;2022:7868391. doi: 10.1155/2022/7868391 (PMC9078787; doi:10.1155/2022/7868391)
Supplement: Supplementary Materials — Supplemental Table 1: information and characterization related to purity results of Perjeta. Supplemental Table 2: information and characterization related to activity results of Perjeta. [file 7868391.f1.docx]

**Supplemental Table1** Information and characterization related to purity results of Perjeta

| Batch | Expiry Date | SEC-HPLC | | NR-CE-SDS | | IEX-HPLC | | | icIEF | | | Unparied Cysteine | | |
| --- | --- | --- | --- | --- | --- | --- | --- | --- | --- | --- | --- | --- | --- | --- |
|  |  | %HMW | %Main | %Fragment | %IgG | %Acidic | %Main | %Basic | %Acidic | %Main | %Basic | %HIC* | %RP* | Kit assay  (mol/mol) |
| H0323B07 | Aug-20 | 0.19 | 99.72 | 2.30 | 97.70 | 20.3 | 65.3 | 14.4 | 26.2 | 68.5 | 5.3 | NA | NA | 0.25 |
| H0324B01 | Aug-20 | 0.18 | 99.72 | 2.56 | 97.44 | 19.6 | 66.0 | 14.4 | 25.6 | 69.3 | 5.1 | NA | NA | 0.21 |
| H0337B01 | Oct-20 | 0.18 | 99.76 | 1.74 | 98.26 | 19.0 | 67.0 | 14.0 | 24.7 | 71.3 | 4.0 | NA | NA | 0.23 |
| H0340B01 | Nov-20 | 0.19 | 99.72 | 2.20 | 97.80 | 20.2 | 66.3 | 13.6 | 27.0 | 68.3 | 4.6 | 10.2 | 15.7 | 0.20 |
| H0371B01 | May-21 | 0.17 | 99.75 | 2.05 | 97.95 | 18.6 | 67.0 | 14.4 | 25.5 | 70.1 | 4.4 | NA | NA | 0.23 |
| H0382B04 | Aug-21 | 0.21 | 99.69 | 2.30 | 97.70 | 21.0 | 66.9 | 12.1 | 26.4 | 68.7 | 4.8 | 10.1 | 15.7 | 0.21 |
| H0388B01 | Sep-21 | 0.20 | 99.68 | 2.16 | 97.84 | 22.6 | 66.3 | 11.2 | 28.0 | 67.7 | 4.3 | NA | NA | 0.21 |
| H0394B01 | Sep-21 | 0.18 | 99.74 | 2.02 | 97.98 | 20.6 | 66.7 | 12.7 | 26.4 | 69.4 | 4.1 | 10.3 | 16.0 | 0.28 |
| H0391B01 | Sep-21 | 0.19 | 99.74 | 2.10 | 97.90 | 19.8 | 65.3 | 14.9 | 26.7 | 68.8 | 4.5 | 10.5 | 16.0 | 0.28 |
| H0389B01 | Sep-21 | 0.21 | 99.69 | 2.06 | 97.94 | 20.9 | 66.2 | 12.1 | 27.4 | 68.1 | 4.5 | NA | NA | 0.21 |
| H0383B01 | Sep-21 | 0.18 | 99.74 | 2.07 | 97.94 | 19.2 | 66.2 | 14.6 | 26.7 | 68.8 | 4.5 | 10.0 | 15.6 | 0.25 |
| H0395B02 | Oct-21 | 0.19 | 99.72 | 2.04 | 97.96 | 20.8 | 66.5 | 12.8 | 26.6 | 69.2 | 4.2 | 10.2 | 15.9 | 0.29 |
| H0396B01 | Oct-21 | 0.18 | 99.72 | 2.04 | 97.96 | 21.0 | 66.2 | 12.8 | 26.9 | 69.0 | 4.1 | NA | NA | 0.32 |
| mean(n=13) | | 0.19 | 99.72 | 2.13 | 97.87 | 20.28 | 66.30 | 13.38 | 26.47 | 69.02 | 4.49 | 10.22 | 15.82 | 0.24 |
| SD(n=13) | | 0.01 | 0.02 | 0.19 | 0.19 | 1.06 | 0.55 | 1.17 | 0.85 | 0.92 | 0.39 | 0.17 | 0.17 | 0.04 |

HMW: high molecular weight impurities; *Using 6 typical batches of Perjeta to detect unpaired cysteine.

**Supplemental Table2** Information and characterization related to activity results of Perjeta

| Batch | Expiry Date | N-glycan Profile | | | FcγⅢa | ADCC (%Relative Activity) | | Fab (%Relative Activity) | |
| --- | --- | --- | --- | --- | --- | --- | --- | --- | --- |
|  |  | %Afucose | %HM | %Galactosylation | %Peak Ⅰ | EC_50_ | Maximum Response | Anti-proliferation | HER2 binding |
| H0323B07 | Aug-20 | 4.2 | 1.1 | 11.8 | 82.61 | 100 | 100 | 80 | 110 |
| H0324B01 | Aug-20 | 4.1 | 1.1 | 9.7 | 84.64 | 96 | 103 | 89 | 106 |
| H0337B01 | Oct-20 | 2.6 | 1.3 | 15.4 | 80.18 | 82 | 86 | 106 | 111 |
| H0340B01 | Nov-20 | 2.4 | 0.8 | 18.5 | 76.84 | 73 | 88 | 78 | 101 |
| H0371B01 | May-21 | 2.1 | 0.7 | 12.4 | 85.38 | 91 | 72 | 105 | 103 |
| H0382B04 | Aug-21 | 3.7 | 1.1 | 9.1 | 86.41 | 84 | 96 | 98 | 102 |
| H0388B01 | Sep-21 | 2.3 | 0.7 | 14.3 | 82.26 | 81 | 74 | 105 | 105 |
| H0394B01 | Sep-21 | 2.1 | 0.8 | 15.2 | 81.31 | 73 | 74 | 98 | 117 |
| H0391B01 | Sep-21 | 2.1 | 0.9 | 15.3 | 80.90 | 71 | 78 | 91 | 116 |
| H0389B01 | Sep-21 | 1.9 | 0.8 | 15.0 | 81.75 | 76 | 70 | 109 | 101 |
| H0383B01 | Sep-21 | 1.8 | 0.7 | 15.4 | 81.62 | 78 | 70 | 115 | 118 |
| H0395B02 | Oct-21 | 2.1 | 0.9 | 15.4 | 81.43 | 76 | 75 | 72 | 108 |
| H0396B01 | Oct-21 | 2.1 | 1.0 | 15.6 | 81.30 | 71 | 80 | 98 | 84 |
| mean(n=13) | | 2.58 | 0.92 | 14.08 | 82.05 | 80.92 | 82.00 | 95.69 | 106.31 |
| SD(n=13) | | 0.84 | 0.19 | 2.63 | 2.43 | 9.50 | 11.54 | 13.02 | 8.99 |

HM: high mannose; ADCC: antibody dependent cell-mediated cytotoxicity; EC_50_: half maximal effective concentration
